# A Web-Based Peer-Patient Navigation Program (Compassionate Online Navigation to Enhance Care Transitions) for Youth Living With Childhood-Acquired Disabilities Transitioning From Pediatric to Adult Care: Qualitative Descriptive Study

**DOI:** 10.2196/47545

**Published:** 2024-02-07

**Authors:** Kristina Marie Kokorelias, Tin-Suet Joan Lee, Mark Bayley, Emily Seto, Alene Toulany, Michelle L A Nelson, Gina Dimitropoulos, Melanie Penner, Robert Simpson, Sarah E P Munce

**Affiliations:** 1 KITE Research Institute Toronto Rehabilitation Institute-University Health Network Toronto, ON Canada; 2 Department of Occupational Sciences and Occupational Therapy, Temetry Faculty of Medicine University of Toronto Toronto, ON Canada; 3 Rehabilitation Sciences Institute Temetry Faculty of Medicine University of Toronto Toronto, ON Canada; 4 Division of Physical Medicine and Rehabilitation University of Toronto Toronto, ON Canada; 5 Institute of Health Policy, Management and Evaluation University of Toronto Toronto, ON Canada; 6 Center for Digital Therapeutics University Health Network Toronto, ON Canada; 7 Toronto General Hospital Research Institute University Health Network Toronto, ON Canada; 8 Department of Adolescent Medicine The Hospital for Sick Children Toronto, ON Canada; 9 Lunenfeld-Tanenbaum Research Institute Sinai Health Toronto, ON Canada; 10 Faculty of Social Work University of Calgary Calgary, AB Canada; 11 Department of Pediatrics Bloorview Research Institute Holland Bloorview Kids Rehabilitation Hospital Toronto, ON Canada; 12 St. John’s Rehab Research Program Sunnybrook Research Institute Sunnybrook Health Sciences Toronto, ON Canada; 13 Rehabilitation Sciences Institute, Temetry Faculty of Medicine University of Toronto Toronto, ON Canada

**Keywords:** youth, patient navigation, web-based intervention, peer support, transition, childhood disability, caregiver, transitional care intervention, social support, usability, program, children, pediatric, disability, digital health, eHealth, web-based support, web-based health

## Abstract

**Background:**

Studies have highlighted significant challenges associated with the transition from pediatric to adult health and social care services for youth living with childhood-acquired disabilities and their caregivers. Patient navigation has been proposed as an effective transitional care intervention. Better understanding of how patient navigation may support youth and their families during pediatric to adult care transitions is warranted.

**Objective:**

This study aims to describe the preferred adaptations of an existing web-based platform from the perspectives of youth with childhood-onset disabilities and their family caregivers to develop a web-based peer-patient navigation program, Compassionate Online Navigation to Enhance Care Transitions (CONNECT).

**Methods:**

A qualitative descriptive design was used. Participants included youth living with childhood-acquired disabilities (16/23, 70%) and their caregivers (7/23, 30%). Semistructured interviews and focus groups were conducted, digitally recorded, and transcribed. Thematic analysis was used to analyze the data and was facilitated through NVivo software (Lumivero).

**Results:**

Participants desired a program that incorporated (1) self-directed learning, (2) a library of reliable health and community resources, and (3) emotional and social supports. On the basis of participants’ feedback, CONNECT was deemed satisfactory, as it was believed that the program would help support appropriate transition care through the provision of trusted health-related information. Participants highlighted the need for options to optimize confidentiality in their health and social care and the choice to remain anonymous to other participants.

**Conclusions:**

Web-based patient navigation programs such as CONNECT may deliver peer support that can improve the quality and experience of care for youth, and their caregivers, transitioning from pediatric to adult care through personalized support, health care monitoring, and health and social care resources. Future studies are needed to test the feasibility, acceptability, usability, use, and effectiveness of CONNECT among youth with childhood-onset disabilities.

## Introduction

### Background

Young people with childhood-onset disabilities (eg, acquired brain injury and cerebral palsy) are living longer than previous generations owing to advances in medical knowledge and clinical management [1,2]. Research and advocacy efforts have focused on ensuring continuous access to health, education, and social services for youth transitioning from pediatric to age-appropriate and developmentally appropriate adult health care services, to support autonomy and maximize independence in society for capable youth [3-7]. Many youths who age out of pediatric services experience a gap in services designed to meet their adult health and social needs [6,8-12]. Furthermore, finding and accessing the appropriate adult care providers and services is often challenging [8,12,13]. Adult health care providers often lack training related to aging with a childhood-onset disability and supporting the unique health and psychosocial needs of young adults [7,14-16].

Youth and young adults living with disabilities acquired in childhood often have chronic health issues that require frequent health care visits, and yet, few receive the comprehensive services and support they need [8,12,17]. Without seamless, accessible, and appropriate services, health concerns may remain poorly managed or undetected, increasing the risk of preventable secondary health complications and comorbidities in young adulthood [18-21], which may lead, in turn, to increased or inappropriate reliance on acute health services (eg, hospitalizations) [7,13,22]. For example, young adults with disabilities aged between 19 and 27 years, including cerebral palsy, spina bifida, and acquired brain injuries, visit physicians and are admitted to the hospital, on average, 9 times more than that among the general population [17,23]. Results from studies conducted in Alberta, Canada, indicate that individuals providing care for children or adolescents with complex care requirements frequently experience feelings of being overwhelmed, fearful, and isolated [24]. Collectively, this evidence highlights gaps in appropriate care for a growing population of transition-age young adults with disabilities (eg, aged 18-30 y) and their caregivers [7]. Closing this gap and ensuring the successful transition from pediatric to adult services is vital to improve the health and well-being of youth and young adults living with disabilities.

Despite the critical importance of successful transition, there is limited evidence about effective transitional care interventions for young adults with childhood-onset disabilities. Most evaluation studies have been descriptive in nature [7], lack rigor in design [25], and often do not use instruments that are valid and reliable for meaningful evaluation [26]. Furthermore, high variability across practice settings and the siloed nature of health and social services have led to issues with transferability to practice settings and community contexts [7]. Previous evidence syntheses in this area, including a systematic review [25], and clinical guidelines [27] have focused mainly on managing chronic medical diagnoses and failed to address the specific and additional needs of the youth with disabilities. It is important to address this gap, as young adults with disabilities may have diverse requirements as they prepare to transition to adult care settings [28,29]. Thus, there exists a pressing need to develop and implement culturally sensitive, accessible, effective, and fiscally sustainable approaches to youth transition. Cost-effective transitions for young adults with childhood-onset disabilities can be expected to have positive, far-reaching impacts on health and social care systems [30].

Although case navigation is a recognized effective transition intervention [31], a recent systematic review found no studies of peer navigation for transition-age youth with childhood-acquired disabilities [7]. Patient navigation emerged in the 1990s as a model of transitional support across health care settings [32,33]. Patient navigation has been defined as a partnership among the patient; family; members of the care team; and patient navigator, who facilitates timely access to health or community resources and fosters self-management and autonomy through education and emotional support [34]. Patient navigators can be peer (lay) navigators (eg, peers with lived experience) or professional navigators (eg, nurses) [32]. Although patient navigation has historically been implemented in the context of adults with cancer, recent programs have focused on children and youth with complex, chronic conditions [35,36]. Patient navigation has been posited as an intervention for youth and young adults with disabilities by reducing barriers to access and integrating various services in a timely, coordinated manner, thus facilitating seamless transitions in care [37]. For example, NaviCare/SoinsNavi is a professional patient navigation center in Canada that is specifically designed to provide support and assistance to children, youth, and their families who are dealing with complex care needs [38]. Patient navigation centers such as NaviCare/SoinsNavi play a crucial role in helping individuals and families navigate the complex health care system by offering guidance, information, and coordination of care [38]. Peer navigation is generally defined as an advantageous interaction between a peer navigator and a patient and traditionally involves a trained peer who provides education and support to a patient to promote recommended health care use behaviors (eg, health screening, attending the recommended care events, and adhering to treatment or follow-up care) with the goal of optimizing care [39,40]. Specifically, through the provision of emotional, informational, and appraisal support, peer navigators can increase patient self-efficacy and, consequently, promote the achievement of recommended health behaviors. However, so far, it is not well known whether and how peer navigation can contribute to the delivery of integrated care for youth with childhood-acquired disabilities transitioning to the adult health care system and community services. Thus, studies of the role that patient navigators may have in assisting during these transitions and specific components of such an intervention are needed.

The NexJ Health Wellness Platform is a web-based platform that has been previously used to build peer navigation programs for adults with chronic illness (eg, cancer [41]). The profile and dashboard display users’ personal information that they wish to share with their circle of care members (eg, peer navigators, health care providers, and families). The profile also includes contact information such that the youth or care team can connect with one another via the platform. The dashboard is adaptable, such that the youth can personalize it with their own background or goals. Points are assigned as individuals meet their goals. The care plan is also where navigators can note any action item that youth should be taking to manage their health (eg, medications to take). Related to this aspect, there is a scheduling feature on the program, which is very similar to a digital calendar, where the clients can set up an appointment with their care team members, who will receive this request and schedule the appointment. Reminders of appointments will be facilitated through the scheduling feature. The health library contains resources provided by the study team that are verified by health care professionals and organized into different categories according to conditions, disabilities, mental health, socialization, mindfulness, and health needs. In phase 1, we received initial ideas about how the platform should be modified to build the Compassionate Online Navigation to Enhance Care Transitions (CONNECT) platform, which are presented as part of phase 2, as described in the following sections.

### Objective

This study aimed to describe the preferred adaptations of this existing web-based platform from the perspectives of youth with childhood-onset disabilities and their family caregivers to develop a web-based peer-patient navigation program called CONNECT. CONNECT aims to be a web-based tool in peer-patient navigation for youth with childhood-acquired disabilities transitioning to adult health care and community services. The development of an evidence-based, patient and family–informed, web-based peer navigation intervention for young adults with childhood-onset disabilities holds the potential to improve transitional care experiences and outcomes [42].

## Methods

### Study Design

We conducted a qualitative descriptive study using semistructured interviews and web-based focus groups [43,44]. Qualitative description is a commonly used methodology in health care research, whereby the primary goal is to describe a complex construct by staying close to the data elicited from the perspectives and in the words of participants with lived experiences [45]. A qualitative descriptive approach is based on individuals’ experiences and points of view—in this case, on peer navigation [38]. We have reported our methods as per the COREQ (Consolidated Criteria for Reporting Qualitative Research) [46].

### Ethical Considerations

The protocol for this study was approved by the (University Health Network Research Ethics Board REB 22-5023). Informed verbal and written consent were obtained from all participants.

### Setting

The study was conducted in Ontario, Canada, where peer navigation has recently emerged as a novel model of pediatric care provincially [47] but where little is known about patient navigation in the context of transition-age youth with disabilities.

### Sampling and Recruitment

A convenience sampling strategy that combined criterion and snowball sampling was used to recruit English-speaking youth, aged between 19 and 30 years with cerebral palsy, intellectual disabilities, or acquired brain injury, and their caregivers [48]. Individuals who were unable to communicate in English were excluded from the study. The recruitment process primarily involved 2 health care organizations that maintain email lists of clients interested in research projects related to youth living with disabilities. The research coordinator used the email list to send invitations to individuals, and in addition, administrators at these organizations verbally promoted the study during group support sessions. Furthermore, social media advertisements were used to reach a wide audience of eligible participants. As part of the snowball sampling approach, at the end of the interview, participants were encouraged to actively inform their peers about the study, facilitating the expansion of our participant network. Eligible participants were subsequently contacted by a study coordinator to obtain informed verbal and written consent. It is important to note that none of the participants had previous affiliations or associations with the research team, ensuring impartiality in data collection.

We initially set a predetermined sample size goal of 15 to 25 participants, which was informed by existing guidance for qualitative research, where the aim is to reach a point of saturation at which new data no longer significantly contribute to the emergence of additional themes or insights [49,50].

### Data Collection and Analysis

#### Overview

Data collection and analysis occurred in 2 phases. Phase 1 aimed to obtain insight about the initial, desired characteristics of a web-based patient navigation program. Before phase 2, we incorporated the findings from phase 1 into the CONNECT program. Phase 2 aimed to obtain feedback about the preferred adaptations of an existing web-based platform to further develop the CONNECT intervention. We also collected information about sociodemographic characteristics to help contextualize the interview data.

#### Phase 1

Semistructured interviews were conducted using an interview guide developed by the research team (refer to Textbox 1 for a sample interview guide). Before the interview, participants were provided with a definition of patient navigation to help orient them to the topic area. Then, 2 experienced qualitative researchers (KMK and TSJL) conducted all interviews over the phone. Each participant completed a single interview ranging between 30 and 75 minutes. Interview were audio recorded and professionally transcribed verbatim. Immediately following each interview, the interviewer wrote reflexive memos about the interview. In total, 61% (11/18) of youths and 39% (7/18) of caregivers participated in the interviews (phase 1).

**Sample interview questions.**

**If money/resources were no object, what would the “ideal” patient navigation intervention look like to you?**

- Not applicable

**Thinking about your experience as you transitioned from pediatric to adult health and social care services, how might a patient navigation program have been helpful when you/your family member transitioned (ie, to adult healthcare, community resources/services)?**

- What benefits do you think such a program would provide to patients and family caregivers that current support, training, resources, programs, services, etc. you receive do not? 

**From your perspective, what are the ideal components of a patient navigation intervention?**

- In what ways, specifically, do you think a patient navigator could help provide education and support?
- What can/should the navigator do?
- What information can/should the navigator provide?
- What information about the program is needed to inform people who are taking part in the intervention?
- What kind of training is needed for the navigators?

Interview data were thematically analyzed, whereby preliminary themes were derived from ongoing data collection and analysis through a coding process [51,52]. First, all transcripts were reviewed for accuracy by author, KMK, who compared the audio files with the transcript. During this process, any preliminary thoughts about the data were recorded. Next, all transcripts were reviewed independently by 3 investigators (KMK, TSJL, and SEPM) and coded using open coding procedures. Discussions around key ideas and codes in the data occurred through a series of weekly meetings to reach consensus on a codebook [51]. This codebook was then applied to the transcripts by 2 researchers (KMK and TSJL), under the guidance of the senior author (SEPM). The coded data were reviewed by the research team, who met at least once weekly to discuss similarities and differences across and within the coded data. This process occurred until preliminary themes were identified. Full-team meetings helped to refine the themes and their content [51]. The full research team comprised content and methodological experts (ie, experts in disabilities, health care transition research, health system research, and qualitative methods). Interviews were stopped when theme saturation was believed to have been achieved, as consistent redundancy was evident in the themes derived from participant experiences [51,53,54]. Data from these interviews were shared with technology developers of the NexJ Health Wellness program to inform the customization of the existing program. An existing web-based program, NexJ Health Wellness, was previously designed to support the monitoring and coaching of chronic diseases in adults.

#### Phase 2

Web-based focus groups were conducted where participants were introduced to the initial features of CONNECT that had been incorporated based on the feedback provided in the interviews (ie, phase 1). However, owing to scheduling difficulties (ie, unable to gather participants on the same day), we also offered participants individual interviews if they preferred. Of the 18 participants who were interviewed and had consented to be contacted for focus groups, 3 (17%) participated in the focus groups and 2 (11%) participated in individual interviews. Some participants who participated in the initial interviews did not participate in the follow-up focus groups or interviews; reasons included the following: their phone or email was not working, and thus, they were unreachable by the research coordinator, and time constraints (eg, work schedules and family obligations). Then, 6 new participants (ie, individuals who did not participate in the original interviews; n=1, 17% caregivers and n=5, 83% youths) were also recruited. Of these 6 participants, 3 (50%) participated in a focus group and 3 (50%) were interviewed individually. These individuals also completed the sociodemographic questionnaires. Overall, 2 focus groups, with 3 participants per focus group, and 5 individual interviews were conducted.

The focus groups lasted approximately 90 minutes and were facilitated by 2 experienced qualitative investigators (KMK and SEPM). A focus group guide (Textbox 2), informed by the preliminary analysis of and reflexive memos from the interviews, was developed by the first and senior authors. During the focus groups, the qualitative investigators strived to ensure that participants had equal opportunity to share their thoughts by using probes to ask individuals their own thoughts. A research assistant took field notes and memos during and after the focus groups [55]. As in phase 1, the research team explored the emergence of new themes as we conducted additional interviews and focus groups. When we reached a point where new interviews did not yield substantially new insights or themes and, instead, reinforced the existing ones, we made the informed decision to conclude the data collection phase.

**Sample focus group guide.**

**From your perspective, what qualities make an effective peer navigator (especially with the view to promoting quality of life and increased participation/integration in the community)? We are defining effective as a program that would help you in promoting quality of life and increased participation/integration in the community.**

- What training should a peer navigator have to be effective?
- How often should the touch-points with peer navigators be?

**Now thinking about the platform you’ve just seen/reviewed, what components/features here would you like/be helpful in your peer navigator program (or the program for your family member)? Why?**

- What components would you dislike/not be helpful (and be helpful to you as a family member)? Why?
- Has the platform captured the issues that are important to you (ie, that you described before)?
- Are there any components not included that you would like to see?

**We are interested in building an online peer navigation program that focuses on compassionate care. Do the features presented here promote the description of compassionate care you hold? Why or why not?**

- If not, what could be added/amplified?

**Is the platform easy to use and understand in terms of its eg, wording and the interface? Why or why?**

- Are there ways that we could improve on these areas?

The individual interviews were conducted by the same 2 interviewers who led the focus groups, using the same guide. All focus groups and interviews were audio recorded and transcribed verbatim. Phase-2 data (ie, interviews and focus groups) were analyzed using the same thematic analysis process as the interviews [51,52]. Following this process, a more critical review of both the interview themes and focus group themes was conducted. Similarly, the coded data from both data sets were combined. Once completed, a side-by-side comparison of the individually coded transcripts was conducted during a team meeting. To help identify the major themes across the data, 3 research team members (KMK, TSJL, and SEPM) led the analysis by individually reviewing the coded transcripts, meeting minutes, and memos. The full investigation team then reviewed the preliminary major themes to reflect about salient ideas, which resulted in full-team discussion and subsequent follow-up discussions to clarify ideas. Hence, investigator and data triangulation were used to ensure the trustworthiness of the data [56,57].

### Positionality of the Research Team

Qualitative researchers are urged to consider how their background and position affect the design, analysis, and reporting of their study [58]. The research team consisted of Canadian researchers with various backgrounds (eg, cultural) and education (eg, trainees, health care professionals, and researchers). Throughout the data collection and analysis process, the research team had frequent discussions to remain cognizant of their own positions and reflect about how these could influence the design of the intervention and the findings. This was the first time the research team had worked with the technology partner. None of the investigators experienced living with cerebral palsy. Throughout the data collection process, we upheld reflexivity by consistently engaging in critical self-reflection and modifying our interview and focus group guides accordingly. This iterative approach empowered us to enhance our questioning techniques and remain responsive to the emergence of new themes and valuable insights.

## Results

### Overview

**Figure 1:**
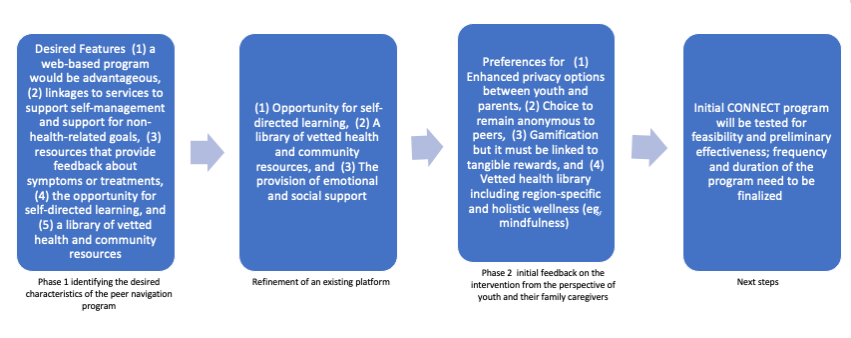
Summary of key findings categorized based on the phase. CONNECT: Compassionate Online Navigation to Enhance Care Transitions.

In total, 24 participants participated in this study, with 5 (21%) participating in both phase 1 and phase 2. Of these 24 unique participants, 8 (33%) were caregivers (all women) and 16 (67%) were youths (n=11, 69% young women; n=5, 31% young men). Most of the caregivers (7/8, 88%) were mothers to a youth with a childhood-onset disability, and a participant was an aunt. Characteristics of the youths and caregivers are reported in Table 1. To secure anonymity, quotations include only the participants’ group (ie, youth or caregiver), sex, diagnosis, and participant ID number. We have synthesized the findings from phase 1 and phase 2 in Figure 1 and Table 2.

**Table 1:** Characteristics of the participants (N=24).

Characteristics			Youths (n=16), n (%)		Family caregivers (n=8), n (%)
**Sex**					
	Female	11 (69)		8 (100)	
	Male	5 (31)		0 (0)	
Age (y)			3 (23)		7 (58)
**Living environment**					
	Urban	15 (94)		8 (100)	
	Rural	1 (6)		0 (0)	
**Highest level of education**					
	Obtained high school	9 (56)		4 (50)	
	Obtained college or university	7 (44)		4 (50)	
**Ethnicity**					
	Asian	4 (25)		2 (25)	
	White	11 (69)		4 (50)	
	South Asian	1 (6)		0 (0)	
	Southeast Asian	0 (0)		2 (25)	
**Primary diagnosis**					
	Intellectual disability	8 (50)		N/A^a^	
	Cerebral palsy	5 (31)		N/A	
	Acquired brain injury	3 (19)		N/A	
**Primary diagnosis of care recipient**					
	Cerebral palsy	N/A		6 (75)	
	Acquired brain injury	N/A		1 (13)	
	Intellectual disability	N/A		1 (13)	

^a^N/A: not applicable.

**Table 2:** Summary of themes.

Phases and themes			Description
**Phase 1**			
	Advantages of web-based programs	In this theme, participants expressed the advantages of a web-based program over in-person support, citing time-saving benefits and independence in navigating web-based resources. Some participants, especially caregivers, acknowledged limited technology knowledge and concerns but saw the potential for enhanced accessibility, particularly for non–health-related goals, through a web-based program.	
	Benefits of peer support	Participants highlighted the benefits of peer support within the proposed program. They emphasized the importance of the peer navigator being trained in individualized, client-centered care and possessing knowledge about regional health and social services. Furthermore, participants stressed the significance of training the navigator in mental health support to aid in transitions from pediatric to adult services.	
	Core components of a navigation program	Participants expressed their desires for several core components of a navigation program: Patient education: Participants emphasized the importance of patient education to enhance their understanding of their condition and treatment options. They believed that this knowledge would empower them with the confidence to actively engage in shared decision-making regarding their health care. Care coordination: Participants stressed the need for care coordination to enable collaborative, patient-centered, and team-based care across various health care settings. This aspect was seen as essential for ensuring seamless transitions in care. Monitoring and coaching: Participants desired remote and mobile support for self-management of their health conditions. They expressed the need for ongoing monitoring and coaching from the research team to help them navigate their health care effectively.	
**Phase 2**			
	Logistical considerations for CONNECT^a^	Participants discussed various logistical considerations for the CONNECT program. They emphasized the importance of specific aspects: Navigator characteristics: Participants expressed a preference for peer navigators with similar life experiences and disabilities. Value of appraisal support: Participants highlighted the need for the navigator to provide appraisal support, including feedback and evaluation. They suggested regular opportunities for participants to provide feedback and suggestions, with input reviewed by trained health care professionals to enhance the program. Necessary infrastructure for accessibility: Participants discussed the importance of accommodating the differences in abilities when using CONNECT.	
	Balancing youth confidentiality with caregiver involvement	Both youth and caregivers highlighted the importance of personalized control over the information shared via CONNECT. Participants believed that navigators could help facilitate discussions with caregivers. Caregivers also wanted control over specific platform functions to prevent unintended actions, suggesting additional confirmation steps for certain actions owing to concerns about unintentional changes.	
	Value of multimodal communication	Participants valued the program’s multimodal communication options, including phone calls, instant messaging, email, and video calls, with the ability to initiate contact themselves. Digital text-based communication was seen as providing fast access to psychosocial support and enhanced privacy for sensitive discussions.	
	Holistic and developmentally appropriate care needs	Participants emphasized the importance of holistic and developmentally appropriate care within the CONNECT program: Developmentally appropriate care: Participants believed that receiving care through CONNECT should consider their unique developmental stages, life events, and personal goals, making their participation in the program meaningful. Point system: Many youth participants did not find value in the point system incorporated into the generic program, especially if points were not linked to tangible outcomes or rewards. Health library: Participants responded positively to the health library, viewing it as a trusted and credible source of health information and comparing it with a more reliable version of a Google search. Forums: Regarding the community forum, participants discussed the issue of anonymity and its impact on their ability to connect with peers. They believed that not remaining anonymous could lead to the potential for meaningful peer connections, providing additional opportunities for mentorship during transitions in care.	

^a^CONNECT: Compassionate Online Navigation to Enhance Care Transitions.

### Phase 1: Development

#### Overview

During the phase-1 interviews, participants described their desires for a web-based patient navigation program. The research team worked with participants during the interviews to identify features for the first iteration of the novel, web-based CONNECT program. Phase 1 consequently identified themes related to the advantages of a web-based patient navigation program facilitated by peer support and the core categories desired in a web-based peer navigation program.

#### Phase 1—Theme 1: Advantages of Web-Based Programs

Several participants explained that a web-based program would be advantageous in comparison with in-person support owing to time-saving benefits related to not needing to travel. Many participants indicated that they would feel comfortable in navigating the web-based navigation program, whereas some caregiver participants highlighted having limited knowledge about technology and associated worries of being unable to support their care recipient:

I think if you can, implement the program using technology as best as you can because that way, I can do it on my own time without finding a drive and I can do all the things that I need to do online independently without assistance. If I do need help, then it’s okay, but I always do it independently when tech. issues come up.

Youth 10; female; cerebral palsy

I guess, not too many families struggle with technology I have, but I don’t know how to fix things. Like, as a single mom, working full-time, I have a child with very, very severe disabilities, not really able to use technology like this. So, I’ll need to learn how to use it to help him.

Caregiver 2; female; cerebral palsy

Although participants reported working with various care providers to support and maintain their health during transitions in care, many were unfamiliar with opportunities for self-management and support for non–health-related goals. Youth believed that that a web-based program would make such services more accessible:

I wish the services, specifically life skills and things like that were more easily accessible, because they’re only in a couple of places right now and you have to have the time available to go to certain sessions wherever they’re happening. And I wish they were more frequent or accessible maybe online and just to be able to talk to people even just for five minutes if you have a question about a goal rather than having to book an appointment a year in advance to see five different people at the same time. It’s not always the best way to get the help that you need, so the program should help with that by being online with one peer.

Youth 8; female; cerebral palsy

#### Phase 1—Theme 2: Benefits of Peer Support

Participants noted that, in addition to being a peer, the navigator should be someone who is trained in individualized, client-centered care with knowledge about the existing health and social services in the region, if possible. Participants also highlighted the importance of training the navigator in mental health support to facilitate the transitions from pediatric to adult services.

Participants highlighted numerous components to be considered for the program. Considerations included resources that provided feedback about disability-related symptoms or treatments; opportunity for self-directed learning; library of vetted health and community resources; and ability to allow for human connection including ongoing communication, compassion, and understanding. Participants validated the notion of peer navigators as being ideal to provide compassionate, appropriate care because they can provide information and emotional support and facilitate health care navigation from a lived experience perspective. Participants also highlighted a general need for support from their navigator with managing personal goals of care and nonmedical transitions (eg, desire to find employment).

Despite consensus on these preferences, participants highlighted that a degree of personalization would be required in the navigator’s approach. Participants believed that the peer navigator is uniquely positioned to provide this individualized support. There was no consensus among participants about the duration for or frequency in which an individual would want to interact with their peer navigator or use the CONNECT program. Thus, participants highlighted that the program should be available for as long as the individual felt that they needed to be enrolled, as transitions can range in time. A participant shared the following:

But maybe 6-12 months or 12-18 months for 2 hours. The reason I say two hours is because there are a lot of things that you have to learn and express, right? If people get the hang of it somewhere, I guess they can go on their own. But if they still have problems with it, or like accessing it even, they can stay longer. Every individual is different, so I would like to see it tailored to their own individual needs.

Youth 2; male; cerebral palsy

#### Phase 1—Theme 3: Core Components of a Navigation Program

Participants described desiring the following: education to improve the understanding of their condition and treatment options for confidence in shared decision-making; care coordination to enable collaborative, patient-centered, team-based care across multiple care settings; and monitoring and coaching to provide remote and mobile support to help self-management, until they were built into a functioning prototype. Participants also described desiring multiple channels and modes of communication to support participants in achieving their health and wellness goals, whereby the peer navigator is the first point of contact for participants.

### Phase 2: Feedback About the Features of the Initial CONNECT Program

In this section, we have outlined the key themes related to the logistics, parental control, multimodal communication, and varied needs for support regarding the use of CONNECT. Figure 1 presents the collective learnings across phase 1 and phase 2.

#### Phase 2—Theme 1: Logistical Considerations for CONNECT

##### Overview

**Figure 2:**
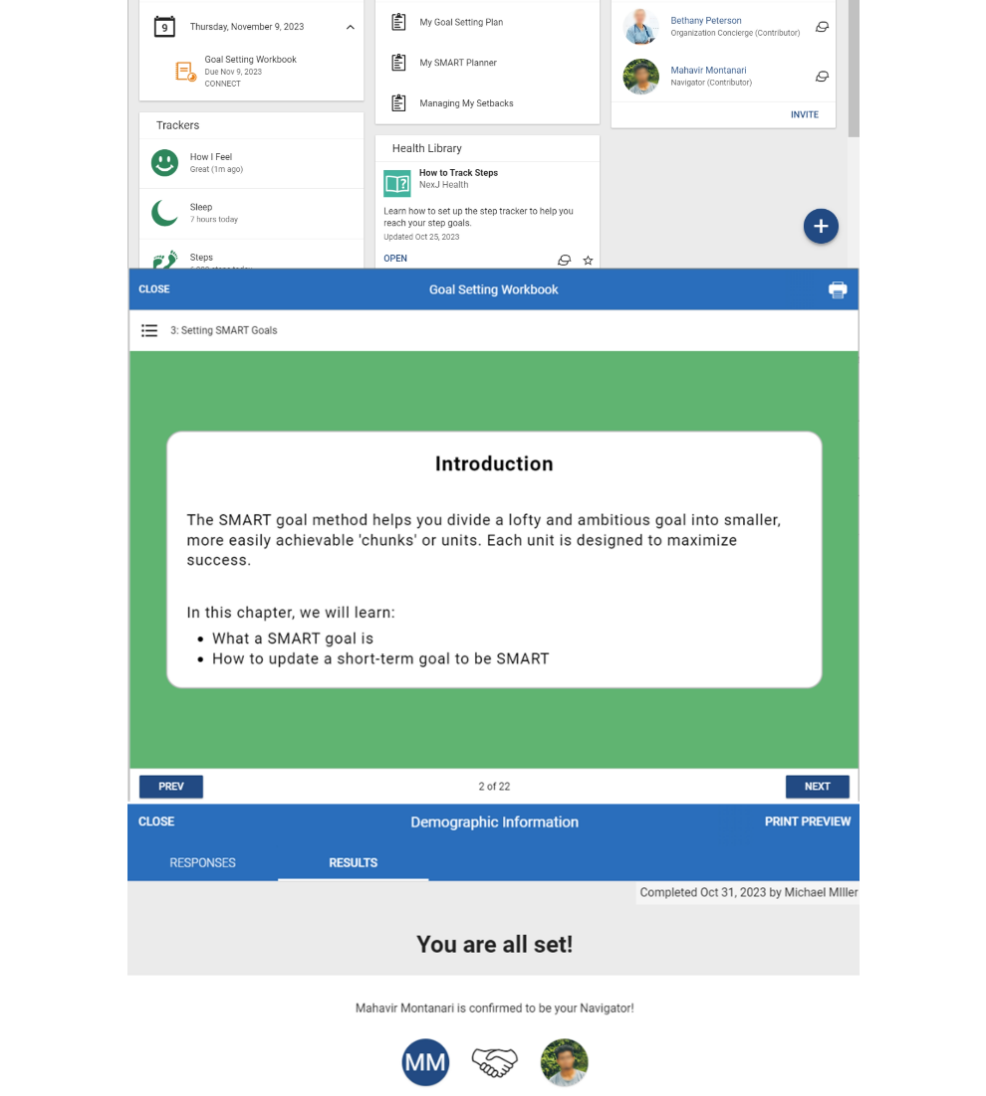
Updated Compassionate Online Navigation to Enhance Care Transitions platform.

Participants described a wide range of logistical considerations related to the CONNECT program. These included the training of the patient navigator, value of appraisal support, and infrastructure needed for CONNECT. We have illustrated these changes in an updated image of the CONNECT system in Figure 2.

##### Subtheme 1: Navigator Characteristics

Participants described their ideal peer navigator as someone with similar life experiences and disabilities. Participants described that they would prioritize someone with these similar experiences over someone of the same age or sex. An individual shared the following:

As a person of colour, and as a self-identifying woman, I would feel more comfortable if someone my age and my demographic were to provide me information, compared to, let’s say just purely an example, of a cis white man.

Youth 19; female; intellectual disability

Another youth participant described this by sharing the following:

It’s just nice to have a person who lived through that experience, and know that somebody has been through it. Actual lived experience is very good versus just a doctor telling you some theoretical things, versus a real person.

Youth 14; male; acquired brain injury

Another caregiver shared the following:

I prefer my son connect with a person who has the same condition. Especially in my son’s case, it’s a little bit different because he’s underweight, he is suffering from dysphasia and he has a G-Tube inserted in his stomach....So, when you have these resources and connect with other persons who have the same condition, it would be very helpful for me and my son both in critical and non-critical situations.

Caregiver 7; female; cerebral palsy

Having time to meet the navigator before receiving care or advice from them was reported as an important facilitator to developing a trusting relationship.

##### Subtheme 2: Value of Appraisal Support

Participants also highlighted the importance of and need for the navigator to assist with appraisal support (ie, evaluation and feedback), particularly in providing feedback to the navigator and other health care providers. A participant shared the following:

I think also maybe just giving them the option to provide feedback and suggestions as needed so maybe having it once a week, or two weeks, or something where a form goes out for them to provide feedback or if they would provide any suggestions. I guess that would be helpful on your side as well when creating it and making the program a bit better so having that going out once every month or so, just so that they know that their suggestions are being heard.

Youth 5; female; intellectual disability

Participants shared that this feedback could be reviewed with trained health care professionals who could provide the navigator with strategies for improvement. Many youths suggested that these strategies should be provided by someone who is trained in mental health care. A youth shared the following:

I think counsellors and therapists, for example, are a very good role model to draw upon these professional qualities from. I think people who are trained in mental health aspects do hold the qualities it takes to create an environment where the participant would feel safe talking about their issues.

Youth 19; female; intellectual disability

##### Subtheme 3: Necessary Infrastructure for Accessibility

Upon reviewing the existing platform, participants highlighted that owing to accessibility concerns, different hardware may be needed to accommodate the differences in abilities when using CONNECT. Examples of hardware mentioned included laptops, desktops, iPads or tablets, and cellular phones. A caregiver described the following:

[My son] cannot use a computer because both of his are closed. Even now, he uses the computer, but I have to open it and set everything up and put the camera in front of him. But also, the iPad, yeah, sometimes is much better because he has hand control movement, so yeah, the iPad is much better for my son, but it’s different for other people.

Caregiver 1; female; has a son with cerebral palsy

Regardless of the technology, almost all participants noted that the device should allow for features such as control over the size of font, brightness, and speech-to-text functions. To serve the multicultural population of Canada, participants emphasized that the program had to be available in English, French, and other languages that may be spoken by users.

Some participants identified the barriers to the use of CONNECT for individuals who may not have access to internet. A participant said the following:

I would say that perhaps having the program in an online program might not work for everyone. They might not be able to access a computer or access the internet. But I think it’s really important to figure out a way to make sure that these individuals are still included in the program and are still able to be supported through the program.

Youth 13; female; acquired brain injury

#### Phase 2—Theme 2: Balancing Youth Confidentiality With Caregiver Involvement

Many youths raised concerns over their parents accessing the information they shared via CONNECT. Youth described that all aspects of the program (ie, communication among the care team and progress posts) should be personalized such that the youth can control who can view their personal health information. Confidentiality came up as an important factor regarding youth feeling comfortable with using CONNECT, particularly in the context of discussing sexual and reproductive health issues or medical concerns with the navigator (eg, impact of the disability on reproductive health). Moreover, youth thought that the navigator could help them with discussions with their caregivers about their role in their care. A youth shared the following:

Thinking of sexual health concerns and like if someone wants their parents to know. Because I know that there are youths who are already basically independent at a very young age, and so they can easily bring up this conversation with their parents because they just have that type of dynamic. Some other youths might have a different dynamic with their parents, such that it’s like, they’ve relied upon them for medical issues and things like that, so they don’t really know how to go about bringing conversations other than, hey, I kind of want to do this. I think in that case, having a navigator would definitely help to express next steps to the youth.

Youth 19; female; intellectual disability

At the same time, caregivers wanted to be able to control the functions their care recipient could access. A caregiver shared the following:

My point is some of the features I don’t want him to play with like canceling appointments. I want to prevent him from doing that on his own. All the features we have on the platform will be absolutely necessary. It’s like in the bank account you have to have it joint with certain people so you can do it. Because if he makes it, sometimes I can’t change it or something, I have to make up the time to make it right back, right? So, that’s what I worry about.

Caregiver 7; female; has a son with cerebral palsy

Another caregiver described that owing to the nature of some disabilities, many of the features on the program should have “an extra layer of clicks or click/confirm options” such as a need to click a second button or confirm button to make the action happen:

My son is 20 years old and he has uncontrolled movement for his hand. Sometimes he pushes the button and makes a mistake. So, what should I do in this case? For my son, he clicks very fast. So, the thing is I want a lock, so both of us to be there, so we can make it available.

Caregiver 1; female; cerebral palsy

#### Phase 2—Theme 3: Value of Multimodal Communication

All participants appreciated that the program allowed for multiple modes of communication. Participants responded favorably to having the option to communicate with the navigator on one’s own terms (ie, phone call, instant messaging, email, and video call), with the contact being initiated by the youth. Simultaneously, participants believed that digital communication (ie, SMS text message) could lead to fast access and more prompt management of their psychosocial issues. Some youths found that not having to communicate verbally provided an added sense of privacy, for example, if they were discussing issues that they did not want others to hear (eg, in the community forum or through messaging their patient navigator). Moreover, caregivers noted that this option may help accommodate youth with nonverbal communication abilities.

Moreover, youth believed that being able to contact the navigator when they wanted could help them better access services for a variety of health and nonhealth issues. For example, a participant shared the following:

My main goal is to be able to be in a place where I can live in an apartment and go to work every day and not necessarily have to think about how my disability will impact me after I’ve already troubleshot it for long enough that I have a routine. So I also just want to talk about that and get support with that. Just living life when something comes up.

Youth 8; female; cerebral palsy

Participants emphasized that the navigator should be available to the youth, caregivers, and care providers beyond standard business hours (eg, Monday to Friday from 9 AM to 5 PM), through a toll-free number, email, or messenger functions on the platform. Participants described wanting to reach a navigator that they trusted with a specific concern or a general need for emotional support during a crisis. Participants suggested having alternative navigators available to support the provision of 24/7 care. A participant stated the following:

Like, because people that will go through health issues, and they need support, but you don’t know when they need the support. You can’t just have it, have someone that’s a registered person be present only from nine to five, or like, I don’t know, eight to four, or something like that. Sometimes going through something right now, like my...like, I’m talking fine right now, but at night I’ll be like, crying in my bed. So, you need to have people there, and someone to talk to at all times. Even at two in the morning, three in the morning. That’s the key, I believe, when you’re building something to support someone. Because our struggles happen all the time, not just during the day and sometimes we have no one who understands us to talk to....So, having a main person from nine, from eight, or whatever, and then having a couple of people at night to, just to...you know, just there. Even though people...even if people don’t need it, you know, you want to be there in case someone wants it, right?

Youth 15; female; intellectual disability

#### Phase 2—Theme 4: Holistic and Developmentally Appropriate Care Needs

##### Overview

Participants indicated that the CONNECT program should provide health education and support that is developmentally appropriate. Youth perceived that receiving care through CONNECT should account for their unique developmental needs, life events, and personal goals, therefore making participation in the program meaningful. Participants operationalized developmentally appropriate care as care that could be personalized to their abilities including up-to-date health information that was written in lay terms and care that could promote self-management.

##### Subtheme 1: Point System

Many youth participants did not value the point system that is a part of the generic program, especially if the points were not linked to outcomes such as a prize. A participant described the following:

I don’t know, a little reward or something tangible, that is motivational, but just having the points itself might not be worth anything to someone who isn’t a child.

Youth 18; female; intellectual disability

##### Subtheme 2: Health Library

Participants responded positively to the health library, often comparing the program with a Google search that was more trusted or credible. A participant described the following:

So, if I had someone recommending things to me that would be incredible. When it’s on an app for healthcare, it’s already you would trust it a lot more as a rule I would think.

Youth 16; female; intellectual disability

However, participants noted that the health library should be expanded beyond physical health, to include mental health and information about accessible hobbies (eg, sport centers), restaurants, and transportation options.

##### Subtheme 3: Forums

Regarding the community forum, participants discussed the issue of anonymity. By not remaining anonymous, participants felt that there would be the potential to meet other peers. The potential to meet more peers with lived experiences was an attractive possibility to many of the youths as it would provide additional opportunities for mentorship through transitions in care. A participant described the following:

I feel like people can actually make friends out of this. Because some people might be going through the same thing, and they might be, eventually, buddies down the road. So, I feel like definitely this is something...especially patient-to-patient. There will be a connection. Like, oh, she or he is going through the same thing as I am. And they will feel like they’re not alone, in case they want to make a decision. I definitely understand why you guys did anonymous, and it's definitely a good option to still keep anonymous, but there should be...if people want to share their name, it’s okay to share their name, so that they can make friends that way.

Youth 15; female; intellectual disability

Participants appreciated the information vetting (eg, using peer navigators as moderators) that would occur in the forums, such that the advice provided by peers would likely be considered legitimate and safe.

## Discussion

### Principal Findings

We have presented the findings from the development process of a web-based patient navigation program that highlighted the preferred adaptations of an existing web-based platform from the perspectives of youth with childhood-onset disabilities and their family caregivers that will be incorporated into a web-based peer-patient navigation program called CONNECT. Participants desired a program that included (1) information about disabilities, (2) self-directed learning, (3) a library of reliable health and community resources, and (4) emotional and social supports. Upon obtaining feedback, we found that participants perceived that CONNECT could help support holistic and developmentally appropriate care needs. Participants also desired a program that was accessible to people with various physical disabilities. Moreover, as with other peer support literature [59-61], we found that for peer navigation to be most meaningful, the navigator should have similar life experiences as the user.

Our findings suggest that youth wanted their personal health information to remain confidential and preferred options of personalized caregiver involvement. Confidentiality is a major factor affecting youth’s decision to access health care services [62]. When health care professionals can assure confidentiality and a trusting relationship, youth are more likely to communicate regarding their needs, engage with follow-up, and develop skills to navigate the health care system [62]. Having a navigator with the same disability and similar life circumstances was viewed as important by participants, as it can help foster trusting relationships. A study of an existing web-based peer navigation program for adult cancer care also found that participants wanted to be matched with a peer navigator who shared common characteristics, particularly the same language and sexual orientation [41]. Optimal Matching Theory, a well-cited theory in the peer support literature that informed CONNECT, suggests that living with a disability or illness creates the need for social support across many aspects of care (eg, physical and occasional) [63]. Matching the support desired with the support provided can enhance outcomes including improved friendship formation, reduced social isolation, and improved mental health [63]. Incorporating simple screening questions regarding language, disability, and sexual orientation may be helpful. It may also be helpful to incorporate specific areas where youth are seeking support, such as emotional, informational, or practical support; their preferred mode of communication; specific modes of web-based delivery; and when and how much the intervention should be delivered. Taken together, these considerations or adaptations may serve to enhance the overall benefits of the CONNECT intervention.

Although there are many definitions of patient navigation [64], implicit in most definitions is the notion that a patient navigator works to meet the health needs of individuals and their families [32]. Our study found that patient navigation should address psychosocial, educational, recreational, and vocational considerations and physical health considerations. In addition to health information, participants also desired information that could facilitate their day-to-day lives such as locating restaurants that are accessible for people with disabilities. Moreover, an important finding from this study was that despite the positive views about having peer support offered in various ways (eg, forum and via the navigator), participants also wanted the information shared and discussed to be vetted by a trained professional. Thus, opportunities for peer navigators to routinely work alongside health care professional navigators may be worth considering in future studies and programs, as current interventions often include solely lay or professional navigators, rather than both [65,66]. For example, youth desired emotional support during times of crises, indicating an example of where care can be better facilitated through trained professionals. Future studies should begin to explore navigation programs that include a combination of professional and peer support and programs that have professional oversight of peer navigators to determine whether and how they can be effectively integrated into transitional support interventions to optimize peer navigation delivery for youth with childhood-acquired brain injury, intellectual disabilities, and cerebral palsy and their families. Our findings provide the preferred requirements for a web-based peer navigation program for youth with childhood-acquired disabilities transitioning from pediatric to adult care. Future studies focused on refining the CONNECT program have the potential to improve the transitional experiences and outcomes of youth living with childhood-acquired disabilities and their families. The age and developmental variations among youth with complex care needs complicate the logistics of patient engagement with the intervention, as tailored approaches are essential owing to diverse cognitive and communication abilities [67,68]. Therefore, addressing these logistical challenges while maintaining a patient-centered, coproduced approach is paramount in the refinement of the CONNECT program.

In a meta-analysis of randomized controlled trials to determine the effects of patient navigation on health care use outcomes, Ali-Faisal et al [69] determined that compared with usual care, patients who received patient navigation were significantly more likely to access health screening and attend a recommended follow-up. Peer-patient navigation was also associated with increased adherence to cancer care follow-up treatment and obtaining early diagnoses [69]. Moreover, data from published studies reporting telehealth solutions for people living with illness or disability suggest the delivery of patient-centered care, relationship building between professionals and patients [68], and supporting medication adherence and health system cost savings [70]. Future directions for this program of research will include evaluating the effectiveness and health economic impacts of an optimized CONNECT intervention in a large-scale, pragmatic, randomized controlled trial. Benefits of the CONNECT program could include increasing participants’ knowledge, skills, and confidence in managing health care transitions and health-related quality of life. The results of a future randomized controlled trial may help determine the potential of CONNECT for wide dissemination and public health impact, if it demonstrates effectiveness.

We acknowledge that implementing CONNECT in real-world clinical practice entails multifaceted challenges. Successful implementation of patient navigation programs within health care systems necessitates planning, funding, multidisciplinary engagement, workflow establishment, communication mechanisms, knowledge user support, appropriate caseload management, and in-kind resource allocation [71]. Thus, to ensure a fit with existing health and social care systems, careful consideration must be given to how the CONNECT system aligns with established health care workflows, processes, and roles [72,73]. Future implementation studies are required to determine who will provide the initial instructions to both users and administrators and are essential for successful adoption. In addition, addressing the provision of ongoing technological support is vital to resolving any technical issues promptly and ensuring seamless operation of CONNECT in the community setting (ie, home) [74]. Thoughtful planning regarding these aspects will play a pivotal role in the effective implementation and sustainability of the system within the complex landscape of health care practices.

### Limitations

This study had some limitations. Most notably, participant selection was biased toward individuals who had high-speed internet and telephone service, as they were more likely to participate in the interviews and focus groups. As such, the needs of individuals living in rural and remote areas, who may be without high-speed internet, and individuals without access to necessary hardware should be considered [75]. Moreover, our study was limited to individuals who could verbally communicate in English, excluding youth with certain communication impairments or disabilities. Moreover, we only recruited individuals with cerebral palsy, acquired brain injury, and intellectual disabilities. All participants in this study were from Ontario, Canada. As such, the preferences and perceptions of the participants may not be transferable to the desires and perceptions of a broad community of youth living with childhood-acquired disabilities [76]. Beginning our intervention development with an existing platform (ie, iterating on an existing platform) may have limited the opportunity to meaningfully co-design the CONNECT program. Finally, our participants explored the existing platform without actual interactions with the peer navigator. By deploying the patient navigation intervention, future studies could also assess its ecological validity [77].

### Conclusions

This study describes the development of CONNECT, a web-based peer-patient navigation intervention for youth with childhood-acquired disabilities to support transitions from pediatric to adult care. Our findings reveal that youth desire receiving peer support from an individual with similar life experiences through multimodal communication techniques and with assurance of confidentiality. At the same time, participants highlighted that for web-based patient navigation to be age appropriate and developmentally appropriate, it must involve trusting relationships and vetted information. Future studies are needed to further refine CONNECT before determining its effectiveness in real-life settings. To the best of our knowledge, this study is the first to explore the desires of youth and their caregivers regarding web-based patient navigation and a codeveloped potential technology solution; however, additional studies are needed to expand the knowledge about the benefits of web-based patient navigation for youth with childhood-acquired disabilities to support transitions from pediatric to adult care.

## Abbreviations

CONNECT: Compassionate Online Navigation to Enhance Care Transitions
COREQ: Consolidated Criteria for Reporting Qualitative Research
